# Cross-Cultural Comparison of Mental Health Between German and South African Employees: Shame, Self-Compassion, Work Engagement, and Work Motivation

**DOI:** 10.3389/fpsyg.2021.627851

**Published:** 2021-06-22

**Authors:** Yasuhiro Kotera, Claude-Hélène Mayer, Elisabeth Vanderheiden

**Affiliations:** ^1^Human Sciences Research Centre, University of Derby, Derby, United Kingdom; ^2^Department of Industrial Psychology and People Management, University of Johannesburg, Johannesburg, South Africa; ^3^Institut für Sprachgebrauch und Therapeutische Kommunikation, Europa Universität Viadrina, Frankfurt, Germany

**Keywords:** cross-cultural comparison, mental health, shame, self-compassion, work engagement, work motivation, Germany, South African

## Abstract

The negative impact of the coronavirus disease outbreak 2019 (COVID-19) on work mental health is reported in many countries including Germany and South Africa: two culturally distinct countries. This study aims to compare mental health between the two workforces to appraise how cultural characteristics may impact their mental health status. A cross-sectional study was used with self-report measures regarding (i) mental health problems, (ii) mental health shame, (iii) self-compassion, (iv) work engagement and (v) work motivation. 257 German employees and 225 South African employees have completed those scales. This study reports results following the Strengthening the Reporting of Observational Studies in Epidemiology (STROBE) guidelines. *T*-tests, correlation and regression analyses were performed. German employees had lower mental health problems and mental health shame, and higher self-compassion than South Africans. Mental health problems were positively associated with mental health shame and amotivation, and negatively associated with work engagement and intrinsic motivation in both groups. Lastly, self-compassion, a PP 2.0 construct, was the strongest predictor for mental health problems in both countries. Our results suggest (i) that German culture’s long-term orientation, uncertainty avoidance and restraint may help explain these differences, and (ii) that self-compassion was important to mental health in both countries. While the levels of mental health differed between the two countries, cultivating self-compassion may be an effective way to protect mental health of employees in those countries. Findings can help inform managers and HR staff to refine their wellbeing strategies to reduce the negative impact of the pandemic, especially in German-South African organizations.

## Introduction

Mental health studies have increasingly gained weight during the past years. Good mental health is associated with high performance, high achievement and the ability to deal with stress (Royal College of Psychiatrists, 2011). Dealing with stress, and coping are important mechanisms working in organizations and of building an increasingly healthy world (Braun-Lewensohn and Mayer, 2020; Mayer and Oosthuizen, 2020). According to the World Health Organization (WHO), 2004, mental health is defined as a state of well-being that enables an individual to realize their abilities, dealing with daily stress. Galderisi et al. (2015) have strongly criticized this definition, pointing out that it is influenced by hedonic and eudaimonic traditions and additionally, that it is influenced by cultural values without being culture-sensitive. Further the authors mention that universal mental health elements need to be identified while culture-specific elements also need to be catered for. This is extremely important with regard to the present cross-cultural study on mental health during the coronavirus disease 2019 (COVID-19). Our understanding of mental health is therefore based on Galderisi et al.’s (2015, p. 232) definition of mental health, regarding mental health as a dynamic state, instead of a fixed one, relating to harmonization with universal values of the world. Moreover, their definition also highlights the relational element of well-being, linking with empathy for others and the body-mind relationship. This mental health definition is viewed as being acceptable for both, the German and the South African socio-cultural context.

Further, mental ill health correlates with “inadequate leadership from supervisors, other difficult social relations in the work environment, or a gap between workplace demands and employees’ competencies” (Burgess et al., 2019, p. 795). Research shows that mental ill-health is globally on the rise, particularly with regard to mental health problems, such as anxiety, depression and stress (Hofmann, 2012). Mental health and ill-health have gained importance also for organizations and workplaces (Mayer and Boness, 2011; Unger and Richter, 2015; Busch, 2016). The national and country-specific approaches to maintaining and developing mental health and well-being in workplaces differ (Gritzka et al., 2020).

In March 2020, the World Health Organization officially declared the spread of the Corona Virus as a pandemic (World Health Organization (WHO), 2020a, b). During COVID-19, mental health deteriorations could be seen in many different contexts (Ripp et al., 2020) and it has been shown previously that pandemics are linked to decreasing mental health and well-being (Anantham et al., 2008; Blakey and Abramowitz, 2017; Shigemura et al., 2020). With regard to COVID-19, particularly the request for social isolation impacted negatively on mental health (Wang et al., 2020). The negative impact of COVID-19 on mental health at work has been reported in the key sectors (e.g., healthcare, infrastructure) and other sectors globally (Sun et al., 2020). COVID-19 brought unprecedented, unforeseen and uncertain changes to many workplaces, leading to stress, anxiety, and depression (Jungmann and Witthölft, 2020). There have been studies which reported the negative impacts of COVID-19 on work mental health (Kotera et al., 2021a), however, only little is known about how this crisis affected workers cross-culturally (Sun et al., 2020). The study at hand aims at closing this gap with regard to the German and the South African cultures with regard to its specific contexts and times. In a crisis, it is highly important how workers (i) deal with uncertainty, (ii) make a decision about their approach to the crisis, and (iii) follow through the approach, which can differ across cultures (Hunynh, 2020). In Germany, although the general population demonstrated improved overall mental health scores (Ahrens et al., 2021), large proportions of employees reported increased stress (43.2%), worries about their future (53.9%) and their family well-being (73.5%) (Kramer et al., 2021). In South African organizations, heightened levels of depression, anxiety, stress and other mental health symptoms were reported (Robertson et al., 2020). These have been further exacerbated by the unstable employment: 2.5 million South African adults have lost their jobs between February and April 2020 (Posel et al., 2021). For this reason, cultural dimensions of Uncertainty Avoidance (how much the society tries to avoid uncertain situations), Long-Term Orientation (their preference to tradition or pragmatism), and Indulgence (how the society values restricting one’s desires) are particularly relevant to workers’ life in COVID-19 (Hunynh, 2020; Jovančević and Milićević, 2020).

Accordingly, this article replicates previous studies run in the United Kingdom, Dutch and Japanese contexts (Kotera et al., 2020) and focuses on two countries that differ in those three dimensions: Germany and South Africa. Based on Hofstede’s six cultural dimensions, those two countries are similar in Power Distance, Individualism, and Masculinity, however, the German society tends to be high on Uncertainty Avoidance and Long-Term Orientation, and low on Indulgence, whereas the South African society tends to be low on Uncertain Avoidance and Long-Term Orientation, and high on Indulgence (Hofstede et al., 2010). In order to discuss both positive and negative factors associated with mental health in these two countries, positive constructs including self-compassion, work engagement and work motivation and negative constructs including negative attitudes and mental health shame will be assessed in this study.

The study at hand focuses on the mental health and well-being in Germany and South Africa, two differing countries in terms of cultures, socio-economic structures. Previous studies have pointed out that countries which are unequal in income also have more unequal well-being (Okulicz-Kozaryn and Mazelis, 2017). Rantanen et al. (2004) have emphasized that promoting health in industrialized and developing countries differs with regard to various aspects, such as stress and occupational health services and that aspect contributes to the importance of the study at hand in which the authors aim at comparing mental health and associated aspects in an industrialized (Germany) and a developmental country (South Africa).

### Mental Health in German Contexts During COVID-19

Generally, mental health in German work context is characterized by extremely low resources and unfavorably high strain (Zok, 2016; Rothe et al., 2017). Research in the German context shows that during the COVID-19 outbreak, mental health and quality of life was directly impacted. Armbruster and Klotzbücher (2020) point out the during the German lockdown, the calling in on telephone-helplines increased by 20% across the country, reflecting in particular the experience of loneliness, anxiety and suicidal ideation. The research further shows that the stronger the lockdown measures were implemented on state levels (Bundesländer), the more did the lockdown impact on the mental health of individuals (Armbruster and Klotzbücher, 2020). Also, Bäumerle et al. (2020) point out that individuals in Germany experienced increased generalized anxiety, depression and distress during the COVID-19 pandemic, while females and younger people reported higher mental burden. Individuals with trust into the government actions and decisions display less anxiety and fear (Bäumerle et al., 2020).

### Mental Health in South African Contexts During COVID-19

In the South African context, organizations are bound to the requirements and provisions of the Health and Safety Act 85 of 1963 (Republic of South Africa, 1993). This requires that organizations and all levels of employees to comply with the conditions contained within the document, including the provision of safety equipment, protective clothing and access to information and communication about safety (Mayer, 2011). Mental health is in South African contexts promoted through health campaigns (Department of Health, 2007). The Occupational Health and Safety Act (Republic of South Africa, 1993) defines health as “free from illness or injury attributable to occupational causes” however, it does not specify physical or psychological illness.

Research has continuously highlighted that South African employees are generally unhealthy due to poor living conditions, lifestyles and diets caused by economic circumstances (Mead, 1998). This unhealthiness brings along high labor turnover rates, high conflict potentials and lower productivity, but also pressure in terms of employee benefit programs (The South African Institute of International Affairs, 2004). Many of the workplaces are experienced as highly stressful in South Africa due to the intra-societal changes within the South African society, decreasing economic stability, political challenges, such as corruption and crime, and financial as well as educational downhills (Mayer and Oosthuizen, 2021 in press). Rothmann (2005) has pointed out that stress is regarded as a high occupational risk, and Sieberhagen et al. (2009) mention that often in the South African context the pathogenic approach to health still remains predominant, and that a more salutogenic and positive psychology-related approach should be considered. Recent research shows that mental ill-health is on the rise in South Africa and that many individuals are unaware that they have a diagnosable disorder which shows a lack of mental health literacy (Kometsi et al., 2019). However, insurance companies warn of increasing disability claims due to psychological, psychiatric and mental disorders (Old Mutual Corporate, 2017).

During COVID-19, South Africa’s national lockdown has introduced serious mental health threats to public mental health according to Kim et al. (2020). Findings from this study show that individuals perceived a risk increase in depression and adults with childhood trauma experiences were more likely to experience significant symptoms for depression (Kim et al., 2020). High rates of severe mental illness and low availability of mental health care was present. Further, the qualitative part of the study showed strong experiences for anxiety, financial insecurity, fear of infection and rumination.

In summary, it can be highlighted that mental health and well-being declined during COVID-19 in South African and German occupational contexts (Hedding et al., 2020; Zacher and Rudolph, 2020).

### Mental Health and Shame

Shame has become a highly important topic in research, as well as in organizational practice (Mayer et al., 2019). According to Kotera et al. (2020) shame about having a mental health problem – which is referred to as mental health shame – can even increase mental health problems. In recent studies (Kotera et al., 2018c, 2019a, 2021b), the authors emphasized that for United Kingdom and Japanese employees, mental health shame was positively related to mental health problems and led to the problem that employees with high mental health shame refrain from attending mental health trainings or seeking of help with mental health problems.

For the German context, Mayer (2019a, b) emphasized that shame in German contexts relates to individual as well as collective issues of shame. Thereby, shame can relate to mental health shame, in particular when individuals experience mental disorders (Mayer, 2019b). In the South African context, Mayer (2019c) has pointed out that shame can easily be experienced when experiencing unemployment or loss of mental health. Kantor et al. (2016) have also highlighted that mental health shame can lead to perceived barriers and facilitation of mental health service utilization, specifically related to stigma, shame and rejection, low mental health literacy, lack of knowledge and treatment-related doubts, fear of negative social consequences, limited resources, time, and expenses.

For the South African context, Egbe et al. (2020) have pointed out that psychiatric stigmatization and discrimination happens in South African contexts and individuals suffering from mental health illness often experience mental health shame which is perpetuated through family members, friends, community care givers and service providers and often leads to a delay in help-seeking. In times of COVID-19, medical and health practitioners have pointed out that there is a lot of shame associated with mental illness during times of COVID-19 and that people should still be reaching out to hospitals and medical and psychological practitioners to overcome shame (Independent Online, 2020).

### Self-Compassion, Work-Engagement and Work Motivation and Constructs Related to Mental Health

A recent study by Sun et al. (2020), which included individuals from over 50 countries, has emphasized that during the COVID-19 crisis, mental health and well-being is viewed as being in the personal responsibility of the individuals across cultures and that emotions, such as calm, love and determination need to be created in organizations to support the well-being of the individuals and employees. In a previous study by Kotera et al. (2020), self-compassion, work engagement and work motivation are associated with mental health outcomes in the Dutch and Japanese work context. This study replicates the study of Kotera et al. (2020) by analyzing data on the same constructs for the German and South African context during COVID-19.

#### Self-Compassion

According to Muris et al. (2016b, p. 787), “Self-compassion can be defined as the tendency to be caring, warm, and understanding toward oneself when faced with personal shortcomings, problems, or failures.” In previous study, researchers have pointed out that self-compassion contributes positively to mental health (Kotera et al., 2020). Individuals with high self-compassion tend to be more aware that hardships are common to the human lives, leading to a balanced view of stressful events (Chishima et al., 2018). Moreover, their non-judgmental openness to present experience allows them to accept negative emotional and cognitive experiences more easily (e.g., Forkus et al., 2019; Anjum et al., 2020). Indeed, a meta-analysis, which examined 79 samples identified a significant relationship between self-compassion and well-being (Zessin et al., 2015). Mayer and Oosthuizen (2020) have pointed out that self-compassion in leaders, particularly in organizational transformations toward the Fourth Industrial Revolution (4IR) is highly important for overall mental health, building a strong sense of coherence and coping mechanisms in workplaces and organizations, particularly in challenging situations. Interventions to foster self-compassion reduce the risk of mental health problems (Muris et al., 2016a) and the experience of shame (Gilbert and Procter, 2006). Montero-Marin et al. (2018) point out that self-compassion differs across cultures. Self-compassion with regard to German and South African work contexts has hardly been compared (Mayer and Oosthuizen, 2020).

Tóth-Király and Neff (2020, p. 9) in their cross-cultural study identified numerous differences in levels of self-compassion: the authors point out that German-speaking respondents tended to indicate the lowest level of self-compassion compared to the other groups. Their results also showed that not only did the respondents who speak different languages differ in their levels of self-compassion, but also cultural differences were evident when comparing the student and community populations, with Germans being in the middle of the field (2020, p. 11).

Individual studies have focused on the concrete effects of self-compassion in Germany. In a Germany-wide study (*n* = 2,404), Körner et al. (2015) found that self-compassion has a mitigating effect on self-cold and can be an important protective factor against depression. Therefore, the authors argue that self-compassion can mitigate in the context of depression—an argument for interventions that promote a self-caring, friendly and forgiving attitude toward oneself. Also, Hilbert et al. (2015) emphasize in their German studies (*n* = 1,158) the psychological and physical health impact of self-compassion, because self-stigmatization and other health risks and burdens can be reduced.

For the South African context, it has been emphasized that self-compassion can support job seekers to deal with negative experiences and to experience lower negative emotions (Kreemers et al., 2018). The authors have further stated that self-compassion decreases negative affect and supports individuals to deal with failure and setback.

#### Work Engagement

With regard to work engagement, this study is based on the conceptualization advocated by Schaufeli et al. (2002). We use this concept of work engagement, since it separates engagement from the related concept of burnout and, furthermore, positions engagement as an independent construct. Based on the authors definition (Schaufeli et al., 2002), work engagement is built on an affective and on a cognitive aspect. Engagement is therefore not only a cognitive component, but at the same based on emotions and feelings (Salanova and Schaufeli, 2008). It is a positive construct, which includes three dimensions, namely vigor, dedication and absorption (Schaufeli et al., 2002). According to other researchers, the concept of work engagement is related to work motivation, however, work engagement is more stable over time (Schaufeli et al., 2002; Mauno et al., 2007). Further on, Hassan and Ahmed (2011) have pointed out that work engagement is associated with positive work attitudes, and Gorgievski and Bakker (2010) have highlighted that higher work engagement is related to better mental health. These findings indicate the relevancy of work engagement to this study. Unsurprisingly, high work engagement has been consistently associated with better mental health in many different occupational populations (Villotti, 2014; Lau, 2017; Veromaa et al., 2017).

Engagement at work is considered one of the relevant factors for quality of life and life satisfaction (Schaufeli and Bakker, 2003; Böhm et al., 2017), which is attributed positive influences on health (Lamers et al., 2012) and positive well-being effects, such as an increased life expectancy (Diener and Chan, 2011). Böhm et al. (2017, pp. 29–31) demonstrated in a study (*n* = 8004) that most of the German employees surveyed (88.7%) were strongly committed to their work, whereby the degree of engagement was influenced, on the one hand by their professional status, and on the other hand by their legal status, age and educational background. Thus, the level of engagement was highest among self-employed and freelancers, and lowest among workers and civil service employees. It was more pronounced among people with permanent contracts than among those with fixed-term contracts. The older the interviewees were, the greater job satisfaction, and higher educational attainment also led to more positive statements regarding work commitment. Lastly, gender differences were minimal. Höge and Schnell (2012) were also able to show that in Germany and Austria meaningfulness and work engagement were correlated strongly.

A study by Mitonga-Monga and Mayer (2020) points out that work engagement of employees is strongly and positively impacted on by a high level of coping, a high sense of coherence level and a low level of burnout in the Democratic Republic of Congo in Central Africa. Employees with a high level or work engagement do positively engage, perform, and are productive. A study on work engagement in South Africa (Barkhuizen and Rothmann, 2006; Sehunoe et al., 2015) shows that there no statistically significant difference on the combined dependent variable, work engagement, between different genders, races, ages or levels of qualification could be found in the South African context. Recent studies within the South African context point out that meaningful work is a strong predictor for work engagement (Ahmed et al., 2016). Stander and Rothmann (2010) have pointed out in the South African organizational context that work engagement increases when employees believe in themselves and when they experience a sense of freedom in their tasks. Psychological empowerment and work engagement in the South African mining sector are also correlated positively (Towsen et al., 2020).

#### Work Motivation

The concept of work motivation deals with the foundational question why employees engage in work (Kotera et al., 2020). Work motivation impacts strongly on the success of organizations (Kanfer et al., 2008). Previous studies (Kotera et al., 2018a, b) on work motivation and mental health have shown that extrinsic motivation is more strongly associated with mental health problems and mental health shame than intrinsic motivation in United Kingdom samples. The significant relationships between work motivation and mental health suggest a need for investigation in our study.

Badura et al. (2018) proved in a study for the German context (*n* = 2000) that it has a positive effect on employees’ health if they experience their work as meaningful. This leads to, for example, higher motivation, lower absenteeism and significantly fewer work-related health complaints. Safe and healthy working conditions and the feeling of doing something meaningful are far more important to employees than a high income. The authors identified the following as relevant intrinsic values: the feeling of being able to perform a meaningful job, a task that is interesting to do, a job where one can work independently and the possibility of having tasks that require a great deal of responsibility. For most employees, personally and socially motivated aspects of their work are relevant to their sense of meaning, which are often not experienced by employees in this way and lead to demotivation (Badura et al., 2018). According to a recent study (Ernst and Young, 2019), the motivation of German employees has significantly decreased recently. The study (*n* = 1500) confirms that only every fourth employee is very satisfied with his work and only 29% are highly motivated. The increased workload and the perceived difficulty in reconciling work and private life were cited as important factors in the decline in work motivation. The appreciation of job performance was named as another decisive factor for motivation in the workplace. Only 60% of the employees indicated that they saw their work at the company as appreciated, and the proportion of women in particular is very low at 55%. At the same time, 47% of men and 34% of the women said that an exciting job motivated them most at work. In comparison, a high salary plays only a minor role and is their main motivation for 20% of the men and 17% of the women. Here, however, not only gender differences but also age differences become obvious, because the group with the lowest motivation or job satisfaction is the group of 30- to 39-year-olds: Only 21% of them are extremely motivated by far the lowest value of all age groups. They want less workload, a better work-life balance and are also willing to accept salary cuts. Rose (2019) was able to demonstrate in his study (*n* = 900) that the motivation of German employees depends above all on company conditions. In his study, he shows that a lack of resources and a lack of career options in particular have a negative impact on motivation, as does the fact that many employees hardly trust the management of their organization. Other factors that inhibit motivation are the lack of regular feedback, both in the form of appreciation and constructive criticism. In this study, too, a gender difference could be demonstrated because women feel a higher level of demotivation, especially due to unqualified managers, a lack of constructive-critical feedback and a stronger experience of the senselessness of their tasks. For men, it is mainly the fear of physical integrity, frustration about being equipped with outdated technology and software and concern about the economic future that inhibits motivation (Rose, 2019, p. 9).

Renard and Snelgar (2017) have pointed out that work motivation within the South African context is strongly connected to three factors, namely personal connection to one’s work, personal desire to make a difference, and personal desire to perform. The same authors have pointed out the intrinsic work motivation was associated positively with work engagement and salary satisfaction and negatively with intention to quit. In a study of Nujjoo and Meyer (2012) findings showed a high intrinsic motivation in South African employees.

Finally, a study of Snelgar et al. (2017) compared German and South African extrinsic and intrinsic work motivation. The results showed preferences for intrinsic motivational factors for the whole sample with higher levels of intrinsic motivation for the South African respondents compared to German respondents. Demographic characteristics played a minor role in determining levels of intrinsic motivation within individuals. The authors emphasize that culture, however, played the biggest role in determining one’s levels of intrinsic or extrinsic motivation.

### The PP 2.0 Perspective in the Context of This Study

The second wave positive psychology (PP 2.0) is commonly regarded as achieving the best in individuals and society, recognizing and using the dark side of human existence through the dialectical principles of yin and yang, instead of liner interpretation of happiness in positive psychology “as usual” (Wong, 2019). As the Chinese/Japanese character for “happiness (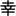)” requires to have “hardship (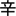)” within it, PP 2.0 does not see the human dark side as a problem, rather it recognizes it as a necessity.

This philosophy is aligned with several aspects researched in this study:

In terms of shame and the transformation of shame, it has been argued recently, that overcoming and transformation of shame – no matter if mental health shame or other forms and experiences of shame – need to take the suffering and negative aspects of shame into consideration and into account to then work through the pain and despair to reach the positive side of shame (Mayer and Vanderheiden, 2019; Mayer et al., 2021).

The foundational assumptions of PP 2.0 (Wong, 2019) are further on reflected in this study with regard to the inclusion of self-compassion in this study because self-compassion also does not deny the dark side; rather it accepts hardship as part of natural human life (e.g., common humanity). Therefore, how strongly self-compassion, a rather PP 2.0 construct, is related to mental health problems, is examined and discussed.

### Aims, Objectives and Value of the Study

This study aims to identify differences and similarities of mental health between German and South African employees to appraise how cultural characteristics may impact their mental health status.

In order to achieve this aim, we will compare mental health and relevant constructs between those two countries, through *t*-tests, correlation and regression analyses. *T*-tests will show the level difference of mental health; correlation analysis will reveal the relationship difference of mental health; and regression analysis will demonstrate the predictor difference of mental health. These differences will be discussed in relation to their cultural differences, namely Uncertainty Avoidance, Long-Term Orientation and Indulgence.

Despite the worldwide impact of COVID-19, the majority of existing studies fail to address how cultures play a role in the dynamics of COVID-19 on work mental health. Resultantly, the implications from these studies may have limited utility when applied to a different cultural group (e.g., the long-term oriented society may agree with a different strategy from the short-term oriented society). Mental health strategies against COVID-19 must consider these cultural differences. Our findings can offer insights into the culture-aware mental health strategies by evaluating the differences of mental health between Germany and South Africa. It is our hope that our findings will facilitate more research into culture and strategies against COVID-19.

## Materials and Methods

### Procedure and Participants

Ethical approval was granted by the Research Review Panel at the European University Viadrina, Frankfurt, Germany. Because this study evaluated the mental health, available mental health services in both countries were introduced throughout the study.

Both German and South African employees were recruited in September 2020 through *Prolific*, a crowdsourcing website whose data quality is regarded comparable to that collected through face-to-face recruitment (Peer et al., 2017). Participants had to be (i) 18 years old or older, (ii) working for an organization in Germany or South Africa at least 3 days a week, and (iii) had been working for at least 6 months. Two hundred and fifty-seven German employees (97 females, 158 males and 1 transgender; age *M* = 34.32, *SD* = 11.01, range 18–70 years old) and 225 South African employees (131 females and 93 males; age *M* = 34.73, *SD* = 11.74, range 19–70 years old) completed the survey written in the English language, taking approximately 15 min on average. Both samples satisfied the required sample size calculated by power analysis (84: two tails, *p*H1(*r*) = 0.30, α = 0.05, power = 0.80, *p*H0 = 0; Faul et al., 2009). Compensation of £1.25 was offered for completing the survey. Compared with the general employee population in Germany (45% female to 55% male), our German sample had more male employees (62% male). Our South African sample had more female employees (58% female), while the national figure was more male-oriented (more than 60% of employees were male; The World Bank, 2020a, b).

In the German sample, 13% of employees worked in higher education (*n* = 33), 9% worked in healthcare and software, respectively (*n* = 24), 8% worked in technical services (*n* = 21), the remaining worked in industries including finance, manufacturing, and hospitality. Many of them (*n* = 162, 63%) had a higher education degree as their highest degree (Bachelor’s *n* = 63, 25%; Master’s *n* = 82, 32%; Doctorate *n* = 17, 7%), 16% had a further education degree (*n* = 40), 20% had a high school diploma (*n* = 51), and 1% had a middle school diploma (*n* = 2).

In the South African sample, 13% worked in finance and insurance (*n* = 29), 11% worked in higher education (*n* = 24), 7% worked in information and technology (*n* = 15), 6% worked in healthcare (*n* = 14), 5% worked in retail (*n* = 12), and the remaining worked in industries such as other education sectors than higher education, construction, and broadcasting. Many of them (*n* = 129, 56%) had a higher education degree as their highest degree (Bachelor’s *n* = 82, 37%; Master’s *n* = 34, 15%; Doctorate *n* = 13, 6%), 17% had a further education degree (*n* = 38), 25% had a high school diploma (*n* = 55), and 1% had a middle school diploma (*n* = 2).

### Instruments

Five scales were used to measure the five variables discussed above.

*Mental health problems* were measured using the Depression Anxiety and Stress. Scale 21 (DASS-21), a shortened 21-item form of the DASS-42 (Lovibond and Lovibond, 1995). Items consider three common mental health problems: depression (7 items, e.g., “I found it difficult to work up the initiative to do things”), anxiety (7 items, e.g., “I was worried about situations in which I might panic and make a fool of myself”), and stress (7 items, e.g., “I tended to over-react to situations”). Each item is responded on a four-point Likert scale (0 = “Did not apply to me at all” to 3 = “Applied to me very much, or most of the time”). DASS-21 has good reliability (α = 0.94 for depression, 0.87 for anxiety, and 0.91 for stress; Antony et al., 1998). For the purpose of this study, the global score was calculated to appraise the level of mental health problems (Lovibond and Lovibond, 1995).

*Mental health shame* was evaluated using the Attitudes Toward Mental Health Problems (ATMHP), comprising 35 items considering (a) negative attitudes, (b) external shame, (c) internal shame, and (d) reflected shame (Gilbert et al., 2007). Negative attitudes (a) relate to an employee’s perception of how their community and family view mental health problems (8 items, e.g., “My community/family sees mental health problems as something to keep secret”). External shame (b) refers to an employee’s perception of how their community and family would perceive mental health problems if they had one (10 items, e.g., “I think my community would see me as inadequate”). Internal shame (c) corresponds to an employee’s perception of how they would see themselves if they had a mental health problem (5 items, e.g., “I would see myself as inferior”). Reflected shame (d) relates to how an employee believes their family would be perceived if the employee had a mental health problem (7 items, e.g., “I would worry about the effect on my family”) and how the employee believes they would feel if a close relative had a mental health problem (5 items, e.g., “I would worry that others would not wish to be associated with me”). Each item is answered on a four-point Likert scale (0 = “Do not agree at all” to 3 = “Completely agree”). All the subscales have good reliability (α = 0.85 for community attitudes, 0.93 for family attitudes, 0.95 for community external shame, 0.97 for family external shame, 0.95 for internal shame, 0.90 for family-reflected shame, and 0.91 for self-reflected shame; Gilbert et al., 2007), and were summed to measure mental health shame (Gilbert et al., 2007).

*Self-compassion* was measured using the Self-Compassion Scale-Short Form (SCSSF; Raes et al., 2011), a shortened 12-item version of the original 26-item Self-Compassion Scale (Neff, 2003b). SCS-SF appraises how kind an employee can be toward themselves in difficult times (Neff, 2003a). Items (e.g., “When something painful happens I try to take a balanced view of the situation”) are answered on a five-point Likert scale (1 = “Almost never” to 5 = “Almost always”). SCS-SF has good reliability (α ≥ 0.86; Raes et al., 2011).

*Work engagement* was measured using the Utrecht Work Engagement Scale-9 (UWES-9; Schaufeli et al., 2006), a shortened nine-item version of the original UWES-17. Three subscales are embedded: vigor (three items, e.g., “At my job, I feel strong and vigorous”), dedication (three items, e.g., “I am proud of the work that I do”), and absorption (three items, e.g., “I feel happy when I am working intensely”). Each item is responded on a seven-point Likert scale (0 = “Never” to 6 = “Always (every day)”). For the purpose of this study, the total score of the UWES-9 was used (α = 0.77 for vigor, 85 for dedication, and 0.78 for absorption; Schaufeli et al., 2006).

Lastly, *work motivation* was evaluated using the Work Extrinsic and Intrinsic Motivation Scale (WEIMS), an 18-item scale appraising three types of motivation—extrinsic motivation, intrinsic motivation and amotivation—based on the Self-Determination Theory (SDT; Tremblay et al., 2009). Amotivation refers to having no motivation to work (three items, e.g., “I don’t know why, we are provided with unrealistic working conditions”). Extrinsic motivation relates to a psychological drive that is stimulated by external instruments such as money and fame (12 items, e.g., “Because I want to be a winner in life”). Lastly, intrinsic motivation occurs when a worker is inherently interested in and passionate about an activity; that activity itself is a reward to them (three items, e.g., “For the satisfaction I experience from taking on interesting challenges”). Each item is scored on a seven-point Likert scale (1 = “Does not correspond at all” to 7 = “Corresponds exactly”). WEIMS subscales have adequate to good reliability (α = 0.80 for intrinsic motivation, 0.70–0.83 for extrinsic motivation, and 0.64 for amotivation; Tremblay et al., 2009).

### Statistical Analyses

After screening for outliers and distribution, measurement invariance tests were conducted to ensure that the scores could be compared between the two groups. Then, scores for mental health problems, mental health shame, self-compassion, work engagement, and work motivation in both groups of employees were compared (Aim 1). Next, correlation analyses were conducted in each sample, to compare correlations among these variables between German employees and South African employees (Aim 2). Lastly, multiple regression analyses were conducted to explore significant predictors for mental health problems in each sample (Aim 3). IBM SPSS version 26.0 was used to perform all analyses.

## Findings

Using the outlier labeling rule (Hoaglin and Iglewicz, 1987), no outliers were detected in both samples. All variables had high reliability (α ≧ 0.70). Descriptive statistics were summarized in Table 1.

**TABLE 1:** Descriptive statistics and *t*-tests: Mental health problems, mental health shame, self-compassion, work engagement and work motivation in German employees and South African employees.

		German employees (*n* = 257)					South African employees (*n* = 225)						
				
Scale	Measured variable (range)	*M*	*SD*	Skewness	Kurtosis	α	*M*	*SD*	Skewness	Kurtosis	α	*t*	*d*
Depression Anxiety and Stress Scale 21	Mental health problems (0–42)	31.47	24.72	1.07	0.54	0.95	41.48	27.41	0.68	−0.10	0.94	−4.19***	−0.38
Attitudes Toward Mental Health Problems	Mental health shame (0–105)	31.97	21.19	0.75	0.08	0.96	37.09	22.37	0.68	0.001	0.96	−2.68**	−0.25
Self-Compassion Scale-Short Form	Self-compassion (1–5)	3.39	0.70	0.03	0.38	0.81	2.85	0.73	0.05	−0.13	0.85	8.19***	0.75
Utrecht Work Engagement Scale-9	Work engagement (0–54)	33.44	11.31	−0.32	−0.62	0.94	34.04	12.17	−0.40	−0.27	0.95	−0.20	−0.02
Work Extrinsic and Intrinsic Motivation Scale	Work motivation												
	Intrinsic motivation (1–7)	4.93	1.48	−0.56	−0.44	0.86	5.08	1.60	−0.63	−0.46	0.86	−0.85	−0.08
	Extrinsic motivation (1–7)	4.83	1.06	−0.30	−0.14	0.84	4.75	1.17	−0.30	−0.26	0.86	0.86	0.08
	Amotivation (1–7)	2.73	1.46	0.56	−0.62	0.77	2.60	1.30	0.53	−0.55	0.70	0.82	0.08

***p < 0.001, **p < 0.01.

Measurement invariance tests were conducted for all variables using principal component analyses (PCA) for both samples (Fischer and Karl, 2019). Bartlett’s tests of sphericity were statistically significant (*p* < 0.001) in all variables, indicating that the data were likely factorizable. PCA identified that in each variable, the same number of components had eigenvalues greater than one (three components for mental health problems, six in mental health shame, two in self-compassion, one in work engagement, intrinsic motivation and amotivation, respectively), except for extrinsic motivation (two in Germans and three in South Africans), between the two groups, which were then confirmed with visual inspection of the scree plot (Cattell, 1966). These findings supported that the scores were comparable between the two groups.

### Comparing the Levels of Work Mental Health (Aim 1)

Because all constructs, apart from self-compassion and extrinsic motivation in German employees, and self-compassion in South African employees, were not normally distributed (Shapiro–Wilk’s test, *p* < 0.05), all data were square-root-transformed to satisfy the assumption of normality. There was homogeneity of variances (Levene’s test for equality of variances, *p* > 0.05). German employees had lower levels of mental health problems (*d* = −0.38; medium size; Cohen, 1988), mental health shame (*d* = −0.25; small size), and higher levels of self-compassion (*d* = 0.75; large size) than South African employees. There were no significant differences in work engagement, intrinsic motivation, extrinsic motivation, and amotivation between the two groups.

### Comparing Correlations (Aim 2)

To evaluate the relationships of mental health problems, mental health shame, self-compassion, work engagement, and work motivation in German and South African employees, Pearson correlations were performed (Table 2). For gender, point biserial correlations were calculated (1 = female, 2 = male).

**TABLE 2:** Correlations between mental health problems, mental health shame, self-compassion, work engagement and work motivation in German and South African employees.

			German employees								
			
			1	2	3	4	5	6	7	8	9
**South African employees**	1	Gender (1 = F, 2 = M)	–	–0.12	–0.09	–0.04	0.16**	–0.06	–0.06	–0.04	0.07
	2	Age	0.06	–	−0.21**	−0.18**	0.18**	0.13*	0.10	–0.09	−0.20**
	3	Mental health problems	−0.19**	−0.32**	–	0.50**	−0.55**	−0.32**	−0.15*	0.004	0.42**
	4	Mental health shame	−0.16*	−0.32**	0.38**	–	−0.36**	−0.18**	–0.07	0.07	0.31**
	5	Self-compassion	0.20**	0.30**	−0.56**	−0.39**	–	0.33**	0.23**	0.01	−0.24**
	6	Work engagement	0.14*	0.25**	−0.34**	–0.12	0.29**	–	0.73**	0.45**	−0.33**
	7	Intrinsic motivation	0.15*	0.16*	−0.27**	–0.05	0.20**	0.72**	–	0.58**	−0.21**
	8	Extrinsic motivation	0.17**	0.14*	−0.17*	0.04	0.16*	0.62**	0.75**	–	0.16*
	9	Amotivation	0.04	−0.17*	0.34**	0.21**	−0.21**	−0.30**	−0.20**	–0.03	–

**p < 0.01, *p < 0.05 (2-tailed).

Our correlation analyses showed that in both workforces, work engagement was associated with all types of work motivation. However, several differences were identified between the two groups. (i) While mental health problems were significantly associated with all variables in South Africans, they were not significantly associated with gender and extrinsic motivation in Germans. (ii) While mental health shame was significantly related to self-compassion, work engagement and amotivation in German employees, it was not significantly related to work engagement in South African employees. (iii) In South African employees, self-compassion was significantly associated with work engagement and all types of motivation, but it was not significantly associated with extrinsic motivation in German employees. (iv) Lastly, all three types of motivation were significantly interrelated to each other in Germans, while extrinsic motivation and amotivation were not significantly related to each other in South Africans.

### Comparing Predictors of Mental Health Problems (Aim 3)

To appraise the relative contribution of each variable to mental health problems, multiple regression analyses were conducted. First, gender and age were inputted to statistically adjust for their effects on mental health constructs (Van Droogenbroeck et al., 2018). Second, data for mental health shame, self-compassion, work engagement, and work motivation were inserted. Adjusted coefficient of determination (Adj. *R*^2^) was reported. Multicollinearity was of no concern (VIF < 10). Results were summarized in Table 3.

**TABLE 3:** Multiple regressions: Mental health shame, self-compassion, work engagement, and work motivation for mental health problems in German and South African employees.

	Dependent variable: Mental health problems							
	
	German employees				South African employees			
		
	*B*	SE_B_	β	95% CI [lower, upper]	*B*	SE_B_	β	95% CI [lower, upper]
**Step 1**								
Gender (1 = F, 2 = M)	–0.54	0.28	–0.12	−1.09, 0.01	–0.78	0.29	−0.17**	−1.36, −0.20
Age	–0.05	0.01	−0.22***	-0.07, −0.02	–0.06	0.01	−0.32***	−0.09, −0.04
Adj. *R*^2^		0.05				0.13		
**Step 2**								
Gender	–0.22	0.22	–0.05	−0.64, 0.21	–0.31	0.25	–0.07	−0.81, 0.19
Age	–0.01	0.01	–0.06	−0.03, 0.01	–0.02	0.01	–0.10	−0.04, 0.002
Mental health shame	0.31	0.06	0.28***	0.02, 0.42	0.16	0.07	0.14*	0.02, 0.30
Self-compassion	–4.13	0.61	−0.36***	−5.32, −2.93	–4.00	0.62	−0.39***	−5.23, −2.77
Work engagement	–0.32	0.15	−0.15*	−0.62, −0.02	–0.18	0.15	–0.09	−0.48, 0.13
Intrinsic motivation	0.98	0.46	0.16*	0.08, 1.88	–0.52	0.53	–0.09	−1.56, 0.52
Extrinsic motivation	–0.72	0.55	–0.08	−1.80, 0.35	0.37	0.68	0.05	-0.96, 1.70
Amotivation	1.19	0.28	0.24***	0.65, 1.73	0.98	0.33	0.17**	0.33, 1.62
Adj. *R*^2^ Δ		0.41				0.27		

***p < 0.001, **p < 0.01, *p < 0.05 (2-tailed).

Self-compassion was the strongest significant predictor for mental health problems in both German and South African employees. Moreover, mental health shame and amotivation were significant predictors for mental health problems, while extrinsic motivation was not. Differences were found where work engagement and intrinsic motivation were significant predictors of mental health problems in German employees, but these were not in South African employees. Table 4 summarized our findings, highlighting the similarities and differences between German and South African employees.

**TABLE 4:** Summary of the findings, comparing mental health of German and South African employees.

	German employees	South African employees
Levels of mental health (Aim 1)	Higher self-compassion	Higher mental health problems and mental health shame
	No significant difference in work engagement and all types of motivation.	
Correlations (Aim 2)	*Similarities*	
	Work engagement was related to all types of motivation	
	*Differences*	
	Mental health problems were related to all variables apart from gender and extrinsic motivation.	Mental health problems were related to all variables.
	Mental health shame was related to self-compassion and amotivation, but not to work engagement.	Mental health shame was related to self-compassion, work engagement and amotivation.
	Self-compassion was related to work engagement, intrinsic motivation and amotivation, but not to extrinsic motivation.	Self-compassion was related to work engagement and all types of motivation.
	Three types of motivation were related to each other.	Three types of motivation were related to each other apart from extrinsic motivation-amotivation.
Predictors (Aim 3)	*Similarities*	
	Self-compassion was the strongest predictor of mental health problems	
	Mental health shame and amotivation were predictors of mental health problems, while extrinsic motivation was not.	
	*Differences*	
	Work engagement and intrinsic motivation were predictors of mental health problems.	Work engagement and intrinsic motivation were not predictors of mental health problems.

## Discussion

In this German-South African comparison study, the levels of, and relationships among mental health problems, mental health shame, self-compassion, work engagement and work motivation were compared. German employees had higher levels of self-compassion than South African employees, while South African employees had higher levels of mental health problems and mental health shame (Aim 1). In South African context, socio-economic pressures are extremely high and is seems to correlate with a higher level in mental health than in the German context which aims at protecting employees and citizen from extreme socio-economic pressures which might provide them with a higher mental health. In both German and South African employee groups, work engagement was significantly related to all three types of work motivation (intrinsic, extrinsic motivation and amotivation). This is could be expected, because previous research has shown, that across cultures, work engagement and work motivation are usually related. Among South African employees, mental health problems were significantly related to all the other variables whereas mental health problems were related to all the others apart from gender and extrinsic motivation among German employees (Aim 2). Lastly, self-compassion was the strongest negative predictors, and mental health shame and amotivation were significant positive predictors of mental health problems in both groups. In contrary, work engagement and intrinsic motivation were significant negative predictors of mental health problems in Germans while those were not significant predators in South Africans (Aim 3). Intrinsic motivation is of very high value in the German context, particularly when it comes to work motivation and it is, accordingly, related to mental health.

The higher levels of mental health problems and mental health shame among South African employees than German employees indicate an alarming state of mental health in South African organizations: despite their challenging mental health, they feel ashamed of having a mental health problem. Indeed, 25% of South African employees (*n* = 1061) who took part in the South African Depression and Anxiety Group (SADAG) survey reported that they have been diagnosed with depression by a professional, yet 32% of them did not tell anybody at work about their mental health status, and 80% of them continued to work with lower performance (South African Depression and Anxiety Group, 2015; Stander et al., 2016). Education and training about mental health may be helpful for South African workplaces to reduce mental health shame and increase mental health awareness, leading to more fact-based conversations and effective recoveries. For example, a recent biological study identified that stress and environmental impacts were more related to depression, rather than whether the person was weak or inadequate (Kobayashi et al., 2020). Knowledge and awareness of mental health in the organization is also crucial as many South African employees did not know how to respond when a colleague revealed mental distress, and did not feel that organizational support was sufficient (Stander et al., 2016). Mental ill health is a taboo in South African contexts. Furthermore, South African employees may benefit from augmenting PP 2.0 constructs to reduce mental health problems, by bypassing mental health shame (Kotera and Ting, 2019; Kotera et al., 2019b, 2018b). For example, self-compassion was strongly associated with mental health problems (Table 2). Implementing self-compassion training may be effective for reducing mental health problems and shame (Gilbert and Procter, 2006). Considering the enormous costs of depression in the country (5.7% of GDP; News24Wire, 2017), these measures should be considered, and implemented rather urgently. Future research needs to evaluate the effects of such interventions on mental health shame and mental health problems of employees in South Africa.

The higher levels of self-compassion among German employees may be explained by the characteristics of German culture; long-term orientation, uncertainty avoidance and restraint (the opposite extreme of indulgence). Cross-cultural research about self-compassion reported that the cultural dimensions of Long-Term Orientation and Uncertainty Avoidance were positively and Indulgence was negatively associated with self-compassion (Montero-Marin et al., 2018). Those cultural dimensions can facilitate civilization, where deferment of gratification and shame are often valued (Hofstede et al., 2010). This may also help explain the development of the German economy; highly civilized work culture of Germany, supported by those cultural characteristics, helped the economy to be advanced, which was also associated with self-compassion. This may mean that this PP 2.0 construct of self-compassion may be relevant to the level of economic advancement. Indeed, a study done in Google reported that psychological safety in the workplace, a relatively similar construct to self-compassion involving compassion toward oneself and colleagues, was strongly associated with work productivity (Rozovsky, 2015). Relatedly, in some contexts, South African workplaces are deemed to be psychologically less safe, associated with a high degree of workplace shame (Mayer and Viviers, 2017; Mayer et al., 2017). As well as a lack of health and safety measures. This shame-sensitive work culture may be expressed in the lower levels of self-compassion among South African employees in our study. Moreover, contrasting findings should be also noted. Uncertainty Avoidance was related to poor mental health in an Italian-Irish study regarding workplace violence (Sommovigo et al., 2018). Indeed, victims of violence in general have poorer mental health, however, evaluation is needed to understand how this cultural dimension can impact mental health constructs. Experiences of violence and crime in the South African culture are extremely high and this might impact strongly on the lower levels of self-compassion. Future research should evaluate how the degree of self-compassion in the workplace plays a role in creating healthy and strong workforce cross-culturally. For example, a workplace wellbeing project to appraise employee mental health including self-compassion adapted to the COVID-19 work style has been proposed (De Angelis et al., 2020). Such adaptable and robust design should be utilized.

Consistent with previous studies explored Japan, Netherlands, and United Kingdom workforces (Kotera et al., 2018a, 2020), mental health problems were positively associated with mental health shame and amotivation, and negatively associated with work engagement and intrinsic motivation in German and South African counterparts too. Considering the strong correlations between mental health problems and mental health shame, directly approaching mental health in these populations may not be effective as that could trigger their mental health shame. This may help explain why their mental health status remains to be challenging despite the raising awareness: employees may be aware of their mental health status but cannot ask for help or engage in mental health training because of shame. Rather than focusing on reduction of mental health problems, augmenting positive constructs such as work engagement and intrinsic motivation may be more effective to German and South African employees as those constructs were negatively associated with mental health problems. Mental health interventions in the workplaces should be implemented considering this strong correlation with mental health shame; refining their positive psychological constructs can bypass their mental health shame. For example, targeting work engagement may be helpful as it was associated with all types of motivation in both samples, leading to higher levels of intrinsic motivation and lower levels of extrinsic motivation and amotivation.

Lastly, despite the cultural and socioeconomical differences between those two countries, self-compassion was consistently the strongest predictor of mental health problems. This may suggest that cultivating self-compassion in the workplace can help improve mental health of employees in both countries. Self-compassion among employees was enhanced through online self-compassion training (Rao and Kemper, 2017), which is feasible in the current pandemic. Such intervention should be implemented and assessed in order to protect challenging mental health of employees in the challenging time. Additionally, this finding may suggest the importance of PP 2.0; self-compassion, a PP 2.0 construct, was the strongest predictor of mental health problems. In both different cultures, recognizing the hardship of one’s life and relating to the meaning was associated with higher wellbeing during the pandemic. This suggests the importance of PP 2.0, especially during the current crisis. Future research should evaluate how PP 2.0 constructs can differentiate themselves from traditional PP constructs in relation to work mental health.

### Limitations of the Study

While this study offered helpful insights, several limitations should be considered. First, participants were recruited through a crowd-sourcing service *Prolific* therefore employees whose mental health was severely poor may not be able to register themselves to this service to take part in research (while noting the compatibility of data quality with face-to-face recruiting). Relatedly, we did not directly examine cultural values at an individual level, and the comparability of the extrinsic motivation was not supported. Second, while the German culture and South African culture illustrated contrasts in the Long-Term Orientation, Uncertainty Avoidance and Indulgence, their socio-economic variables differed as well. As discussed above, these differences might explain part of the differences in mental health between them, which is out of the scope of this study. However, it is common to directly compare psychological variables between two countries in this type of study (e.g., Pflug, 2009; Kotera et al., 2020, 2021c). Third, this study used self-report measures, which therefore might limit the accuracy. This was why our study compared relativity of the constructs in correlation and regression analyses, however future research should use biological measures or implicit tests to more accurately compare those two countries. Additionally, this study did not consider other common wellbeing variables, because their relationships with mental health shame was not reported. For example, PsyCap has been reported highly relevant to workplace wellbeing (Luthans and Youssef-Morgan, 2017). Moreover, considering the current pandemic, evaluating trauma-related coping in relation to the mental health variables may yield practical findings, as such coping can protect employees from mental distress (Sommovigo et al., 2018). Future research should include these variables to further explore pertinent factors for mental health shame and other variables explored in this study. Lastly, longitudinal studies are recommended in order to elucidate the causal direction of the associations identified in this cross-sectional study.

## Conclusion and Recommendations

Workplace mental health has gained increasing importance to today’s world, particularly during the COVID-19 pandemic. While the general impacts of COVID-19 on work mental health have been reported, how cultures can play a part in their mental health remained to be explored. This study compared the levels of, and relationships among mental health problems, mental health shame, self-compassion, work engagement and work motivation between German employees and South African employees. German employees had lower levels of mental health problems and shame, and higher levels of self-compassion than South African employees. Self-compassion, a PP 2.0 construct was deemed most strongly related to mental health in both workforces. The findings suggest that German culture’s long-term orientation, uncertainty avoidance and restraint may explain these differences in mental health and self-compassion scores. Moreover, self-compassion being the strongest predictor of mental health in both countries indicates the importance of PP 2.0 to German and South African workplaces in order to overcome mental health challenges caused by COVID-19. Future research should evaluate the effects of specific self-compassion training that can be practiced by busy workers in both countries, and discuss what type of training would fit better with their own culture. As the mental health impacts of COVID-19 remain to expand, both German and South African organizations should consider self-compassion as an effective means to overcome this unprecedented challenge.

## Data Availability Statement

The raw data supporting the conclusions of this article will be made available by the authors, without undue reservation.

## Ethics Statement

The studies involving human participants were reviewed and approved by Research Review Panel at European University Viadrina. The patients/participants provided their written informed consent to participate in this study.

## Author Contributions

YK contributed to the study concept and design and statistical analysis. YK and C-HM contributed to the acquisition and preparation of the dataset. All authors contributed to the interpretation of the data, drafting of the manuscript, and critical revision of the manuscript for important intellectual content. All authors had full access to all of the data in the study and takes responsibility for the integrity of the data and the accuracy of the data analysis. All authors saw and agreed on the final manuscript as well as the decision to submit for publication.

## Conflict of Interest

The authors declare that the research was conducted in the absence of any commercial or financial relationships that could be construed as a potential conflict of interest.
